# Hearing loss in Norwegian adults with achondroplasia

**DOI:** 10.1186/s13023-021-02095-7

**Published:** 2021-11-04

**Authors:** Svein O. Fredwall, Björn Åberg, Hanne Berdal, Ravi Savarirayan, Jorunn Solheim

**Affiliations:** 1 TRS National Resource Centre for Rare Disorders, Sunnaas Rehabilitation Hospital, 1450 Nesodden, Norway; 2 Faculty of Medicine, Institute of Clinical Medicine, University of Oslo, Oslo, Norway; 3 Department of Hearing, Lovisenberg Diaconal Hospital, Oslo, Norway; 4 Victorian Clinical Genetics Services, Murdoch Children’s Research Institute, University of Melbourne, Parkville, Australia

**Keywords:** Hearing loss, Audiometry, Tympanometry, Impedance audiometry, Craniofacial abnormalities

## Abstract

**Background:**

Achondroplasia is the most common form of disproportionate skeletal dysplasia. The condition is caused by a mutation in the *FGFR3* gene, affecting endochondral bone growth, including the craniofacial anatomy. Recurrent otitis media infections, chronic middle ear effusion, and hearing loss are common in children with achondroplasia, but few studies have investigated hearing loss in adults with this condition.

**Objectives:**

This population-based study investigated the prevalence, severity, and type of hearing loss in Norwegian adults with achondroplasia.

**Methods:**

We collected data on 45 adults with genetically confirmed achondroplasia: 23 men and 22 women, aged 16–70 years. All participants underwent a comprehensive audiologic assessment, including medical history, pure-tone audiometry, speech audiometry, and impedance audiometry. According to the Global Burden of Disease classification, pure-tone average ≥ 20 decibel hearing level (dB HL) was considered clinically significant hearing loss.

**Results:**

Insertion of ventilation tubes had been performed in 44% (20/45) of the participants, 49% (22/45) had a history of adenoidectomy, while 20% (9/45) used hearing aids. Hearing loss in at least one ear was found in 53% (24/45) of the participants; in 57% (13/23) of the men and 50% (11/22) of the women. In the youngest age group (age 16–44 years), 50% (14/28) had hearing loss, although predominantly mild (20–34 dB HL). An abnormal tympanometry (Type B or C) was found in 71% (32/45) of the participants. The majority (15/24) had conductive hearing loss, or a combination of conductive and sensorineural hearing loss (8/24).

**Conclusions:**

Adults with achondroplasia are at increased risk of early hearing loss. Our findings underline the importance of a regular hearing assessment being part of standard care in achondroplasia, including adolescents and young adults. In adult patients diagnosed with hearing loss, an evaluation by an otolaryngologist should be considered, and the need for hearing aids, assistive listening devices, and workplace and educational accommodations should be discussed.

**Clinical trial registration** ClinicalTrials.gov identifier NCT03780153.

## Introduction

Achondroplasia is the most common form of disproportionate skeletal dysplasia, affecting more than 250,000 individuals worldwide [1]. The condition is caused by a mutation in the Fibroblast Growth Factor Receptor 3 (*FGFR3*) gene, affecting endochondral bone growth, including the craniofacial anatomy [2]. Individuals with craniofacial syndromes are at high risk of middle ear disease and hearing loss [3–5]. In achondroplasia, midface hypoplasia, short Eustachian tubes, small pharynx, and the relative enlargement of tonsils and/or adenoids, are predisposing factors for recurrent upper airway infections and chronic middle ear effusion [3, 6–9]. Prior studies on children with achondroplasia have reported that up to 70% have experienced acute or recurrent otitis media [9–11], giving increased risk of persistent middle ear disease and permanent hearing loss [5, 12]. Hearing loss has been reported in 50–70% of children with achondroplasia [6, 7, 9, 13]. However, few studies have investigated hearing loss in adults with this condition, and no population-based studies exists [6, 14].

Tunkel et al. reported that 55% out of 28 adults with achondroplasia screened at a patient support meeting failed hearing screening in one or both ears, and 39% had abnormal tympanometry [13]. Hunter et al. reported that 38% of the adult study population (n = 43) had hearing loss, but audiograms were not available in all [9]. Glass et al. conducted pure-tone audiometry and tympanometry in 28 adults with achondroplasia [15]. Hearing loss was found in 61% of the participants, of whom the majority had conductive hearing loss, and 51% had an abnormal middle ear function [15].

According to the Global Burden of Disease Study 2019, hearing loss was the third largest cause of disability worldwide, with an overall global prevalence of 19% [16]. In Europe, the overall prevalence was 14%. The hearing loss was largely caused by ageing, and 62% of those with impaired hearing were older than 50 years [16]. In the large population-based Norwegian HUNT4 Hearing study (n = 28,339), conducted between 2017 and 2019, 3% in the age group 20–44 years had hearing loss, 15% in the age group 45–64 years, and 64% in the age group over 64 years [17]. In children, hearing loss can cause speech delay and learning problems, and educational attainments might be compromised [5, 18–20]. In adults, hearing loss impairs communication, and may cause social isolation, withdrawal from society, reduced employment opportunities, limitations in activity, and reduced quality of life [16, 18, 20–22].

Currently, there are no widely accepted recommendations for hearing assessment in adolescents or adults with achondroplasia. In the recently revised recommendations on Health Supervision for People with Achondroplasia (2020), provided by the American Academy of Pediatrics, the recommendation of a formal routine hearing assessment in children, ideally on an annual basis, has been extended to also include adolescents and adults [23]. Other guidelines recommend a routine assessment of hearing in early childhood at the time of diagnosis and at age 5 years, and further assessment if there is a speech delay, suspicion of hearing difficulties, or signs or symptoms of middle ear disease [10, 12].

In the present study, we conducted a comprehensive audiologic assessment to investigate the prevalence, severity, and type of hearing loss in Norwegian adults with achondroplasia.

## Methods

### Study design and population

This cross-sectional study was part of The Norwegian Adult Achondroplasia Study, a population-based study conducted between 2017 and 2019 on community-dwelling, Caucasian adults, aged 16 years or older, living in Norway [24]. Details of the recruitment process, and inclusion and exclusion criteria in The Norwegian Adult Achondroplasia Study, have been described elsewhere [24].

### Data collection and clinical measurements

A medical history was obtained in a face-to-face interview, including history of otitis media, upper airway infections, prior upper airway surgery, prior diagnosis of impaired hearing, and the use of hearing aids. Participants were also asked whether they received any regular follow-up for hearing loss. Demographic and anthropometric data were collected from The Norwegian Adult Achondroplasia Study [24]. All hearing measurements were conducted at the hearing central at Lovisenberg Diaconal Hospital by an experienced audiologist (JS). The measurements were performed under standardized conditions, in accordance with current international reference standards, and included pure-tone audiometry, speech audiometry, and impedance audiometry. Before the tests, all participants underwent an otomicroscopic examination with photo documentation by an otolaryngologist (HB) or an otosurgeon (BÅ). An OTOsuite audiometer (by OTOmetrics AS) was used for hearing tests. Titan Imp 440 (by Interacoustics AS) was used for impedance audiometry.

#### Pure-tone audiometry

Air- and bone-conducted pure-tone thresholds were obtained to measure hearing sensitivity, and provide diagnostic information regarding severity, type and configuration of hearing loss. Pure-tone thresholds were measured separately for the left and right ear and in accordance with the International Organization for Standardization (ISO 8253-1:1989). Pure tones for the frequencies 0.25, 0.5, 1, 2, 3, 4, 6 and 8 kHz were obtained for the air conduction test, and the frequencies 0.25, 0.5, 1, 2, 3 and 4 kHz for the bone conduction test. *Better ear* refers to the subject’s better ear when comparing the pure tone average (PTA) of the frequencies 0.5, 1, 2 and 4 kHz of the left and the right ear (BE PTA4).

#### Speech audiometry

Speech audiometry was carried out to assess speech intelligibility and discrimination, and to crosscheck the results obtained by pure-tone audiometry. A Norwegian Speech Audiometry set (“HiST speech audiometry”) was used for speech recognition measurement [25]. The test set consists of three-word utterances of the form numeral-adjective-noun. Speech audiometry was performed according to current reference standards (NS-EN ISO 8253-3/ISO 8253-3:1996).

#### Impedance audiometry

Impedance audiometry (tympanometry and stapedius reflex test) was conducted to determine the pressure in the middle ear, evaluate acoustic reflex pathways and tension of the tympanic membrane. The test was also used to distinguish a conductive from a sensorineural hearing loss.

The tympanometry results were displayed as a graph showing compliance, gradient and pressure according to normative values, and was categorized as Type A (normal), Type B (flat, clearly abnormal) or Type C (significantly negative pressure in the middle ear, possible indicative of pathology) [26]. For further statistical analyses, the tympanometry categories were dichotomized to normal (Type A) or abnormal (Types B or C).

Stapedius reflex test was measured both ipsilateral and contralateral. We measured the contraction and thresholds for activation of the stapedius in response to stimuli of 0.5, 1, 2, and 4 kHz, at intensities of 70–115 decibel (dB) sound pressure level. The outcome was dichotomized to normal or abnormal for one or both ears [27].

### Type of hearing loss

Type of hearing loss was categorized as (a) normal hearing/no hearing loss, (b) conductive hearing loss, (c) sensorineural hearing loss, or (d) mixed hearing loss. A conductive hearing loss was defined as air-bone gaps larger than ≥15 dB at one frequency or ≥10 dB at two frequencies [28]. A sensorineural hearing loss was defined as a hearing threshold ≥ 20 dB hearing level (dB HL), but with an air-bone gap <15 dB at one frequency or <10 dB at two frequencies. Mixed hearing loss was defined as a combination of conductive and sensorineural components.

### Hearing thresholds and severity of hearing loss

Severity of hearing loss was defined according to the Global Burden of Disease classification: normal hearing: < 20 dB HL; mild hearing loss: 20.0–34.9 dB HL; moderate hearing loss: 35.0–49.9 dB HL; moderately severe hearing loss: 50.0–64.9 dB HL; severe hearing loss: 65.0–79.9 dB HL; profound hearing loss: 80–94.9 dB HL; complete or total hearing loss: ≥ 95 dB HL [22, 29]. Disabling hearing loss was defined as ≥ 35 dB HL in the better ear (BE PTA4) [18, 30].

### Statistical analyses

Descriptive statistics are presented as frequencies (n) with percentages (%) for proportions, or means with standard deviation (SD) for continuous variables. Independent sample t-tests were used to compare differences between continues variables. Continuity corrected chi-squared tests were used for comparing proportions (using the “prop. Test” R function). Confidence intervals for proportions was found using the Exact Binominal Test (applying the “binom.test” R function). Statistical significance was set to p < 0.05 (two-sided). Statistical analyses was performed using the Statistical Package for Social Sciences (SPSS) version 26 (IBM Corp., Armonk, New York), and R version 3.6.1.

## Results

### Study population and medical history

Out of the 50 participants in The Norwegian Adult Achondroplasia Study, 45 were included in the present study: 23 men and 22 women. Those five not included were all visited in their homes due to impaired health [24], making standardized hearing measurements impossible. All participants had genetically confirmed achondroplasia [24]. Mean age was 38 years, with a range from 16 to 70 years, 73% (33/45) were single/lived alone, and 62% (28/45) were currently working or were students (Table 1). Insertion of ventilation tubes had been performed in 44% (20/45) of the participants, 49% (22/45) had a history of adenoidectomy, while 20% (9/45) used hearing aids. Nine participants (20%) reported to have regular follow-up by an otolaryngologist. Except for height, there were no significant differences in the characteristics between men and women with achondroplasia (Table 1).

**Table 1:** Characteristics of adult participants with achondroplasia

Variables	All (n = 45)	Men (n = 23)	Women (n = 22)	P value ^a^
Age, years, mean (SD)	37.7 (16.6)	39.1 (17.6)	36.2 (15.8)	0.56
Height, cm, mean (SD)	133.1 (9.1)	137.0 (8.7)	129.1 (7.8)	0.002

Variables	N (%)	N (%)	N (%)	
Single/living alone	33 (73)	17 (74)	16 (73)	1.0
Working ^b^ or student	28 (62)	13 (57)	15 (68)	0.54
Medical history				
Acute otitis in childhood	37 (82)	17 (74)	20 (91)	0.24
Acute otitis in adulthood	10 (22)	5 (22)	5 (23)	1.0
History of ventilation tubes	20 (44)	11 (48)	9 (41)	0.77
Adenoidectomy	22 (49)	12 (52)	10 (46)	0.77
Currently using hearing aid	9 (20)	4 (17)	5 (23)	0.72

*SD* standard deviation

^a^Independent sample t test for continuous variables, and Fisher’s exact test for proportions

^b^Working full time, or part time ≥ 50%

### Hearing loss in adults with achondroplasia

Hearing loss (≥ 20 dB HL) in at least one ear was found in 53% (24/45) of the participants: in 57% (13/23) of the men and 50% (11/22) of the women (difference 7%, 95% confidence interval − 27 to 40%, p=0.89). Unilateral hearing loss was found in 11% (5/45), while 42% (19/45) had bilateral hearing loss (Table 2). The majority (18/24) had mild hearing loss (20–34 dB HL). In the youngest age group (age 16–44 years), 50% (14/28) had hearing loss, although predominantly mild. The speech audiometry findings were in accordance with the findings assessed by pure-tone audiometry.

**Table 2:** Hearing loss in adults with achondroplasia, by age groups

Age groups, years	All	16–44 y	45–64 y	> 64 y
	(N = 45)	(n = 28)	(n = 14)	(n = 3)
	N (%)	N (%)	N (%)	N (%)
Hearing loss in at least one ear^a^				
Mild (20–34 dB)	18 (40)	12 (43)	6 (43)	0 (0)
Disabling (≥ 35 dB)	6 (13)	2 (7)	1 (7)	3 (100)
Unilateral hearing loss^a^				
Mild (20–34 dB)	3 (7)	3 (11)	0 (0)	(0)
Disabling (≥ 35 dB)	2 (4)	2 (8)	0 (0)	(0)
Hearing loss in the better ear^b^				
Mild (20–34 dB)	15 (33)	9 (32)	6 (43)	0 (0)
Disabling (≥ 35 dB)	4 (9)	0 (0)	1 (7)	3 (100)
Abnormal tympanometry^c^				
Unilateral	8 (18)	5 (18)	3 (21)	0 (0)
Bilateral	24 (53)	12 (43)	9 (64)	3 (100)
Abnormal stapedius reflex^d^				
Unilateral	5 (11)	3 (11)	2 (14)	0 (0)
Bilateral	27 (60)	16 (57)	8 (57)	3 (100)
Type of hearing loss				
Conductive	15 (33)	12 (43)	3 (21)	0 (0)
Sensorineural	1 (2)	1 (4)	0 (0)	0 (0)
Mixed	8 (18)	1 (4)	4 (29)	3 (100)

*dB* decibel

^a^Based on PTA over 0.25, 0.5, 1, 2, 4, 6 and 8 kHz

^b^Based on PTA over 0.5, 1, 2 and 4 kHz (BE PTA4)

^c^Defined as Type B or C

^d^Absent or reduced contraction of the stapedius muscles

Hearing loss in the better ear (BE PTA4 ≥ 20 dB HL) was found in 42% (19/45) of the participants: in 32% (9/28) of the youngest age group, and 50% (7/14) of those aged 45–64 year. The majority (15/19) had mild hearing loss, while four had disabling hearing loss (hearing threshold ≥ 35 dB HL).

### Impedance audiometry

An abnormal tympanometry (Type B or C) in at least one ear was found in 71% (32/45) of the participants (Table 2), of whom 18% (8/45) were unilateral. All with an abnormal tympanometry also had hearing loss. An abnormal stapedius reflex in at least one ear were found in 71% (32/45) of the participants, of whom 11% (5/45) were unilateral (Table 2).

### Type of hearing loss

Of those with hearing loss, 63% (15/24) had a conductive hearing loss, 4% (1/24) had a sensorineural hearing loss, and 33% (8/24) had a mixed hearing loss (Table 2). One male participant had profound unilateral hearing loss (hearing threshold 84 dB HL) due to previous acute trauma to the ear. None of the participants reported prior occupational noise exposure.

## Discussion

In this population-based study, over half of Norwegian adults with achondroplasia had hearing loss (≥ 20 dB HL) in at least one ear, including half of the youngest age group (age 16–44 years). There was no considerable difference between men and women. The majority had conductive hearing loss. An abnormal tympanogram (Type B or C) in at least one ear was found in over 70% of the participants.

To our knowledge, this is the largest clinical study to date investigating hearing loss in adults with achondroplasia and performed under standardized conditions. Our findings are consistent with prior studies, having reported a prevalence of hearing loss of about 55–60% in adults with achondroplasia [13, 15].

**Fig. 1:**
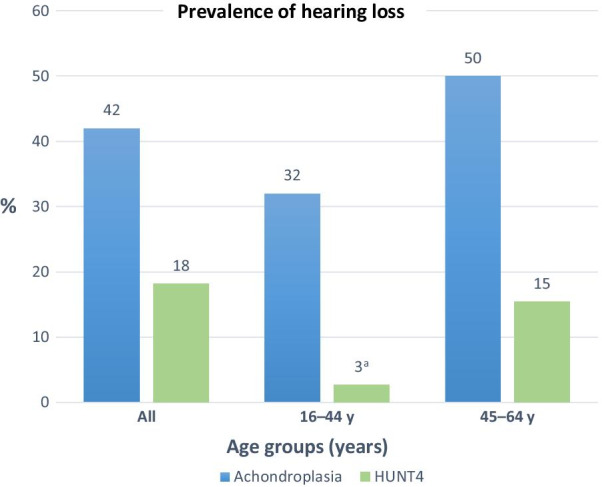
The figure displays the prevalence of hearing loss (≥ 20 dB hearing level) in participants with achondroplasia, by age groups, compared to the prevalence reported in the population-based Norwegian HUNT4 Hearing Study [17]. The prevalence of hearing loss was considerably higher in participants with achondroplasia compared to HUNT4 across all age groups, and particularly in the youngest participants. For achondroplasia participants > 64 years of age (n = 3), all had disabling hearing loss (≥ 35 dB HL), but due to the small sample size, comparison to HUNT4 was not possible for this age group. ^a^Participants with achondroplasia in the age group 16–44 years were compared to the age group 20–44 years in HUNT4

We compared our data to the Norwegian HUNT4 Hearing study, both based on BE PTA4 [17]. The prevalence of hearing loss was considerably higher in the achondroplasia study population compared to HUNT4 across all age groups, and particularly in the youngest age group (Fig. 1). In participants with achondroplasia aged 16–44 years, 32% had hearing loss, compared to 3% in the HUNT4 population (20–44 years). In the age group 45–64 years, 50% of the achondroplasia participants had hearing loss, compared to 15% in HUNT4. Only three achondroplasia participants were over 64 years, all with disabling hearing loss, compared to 18% with disabling hearing loss in this age group in the HUNT4 study [17].

Hearing loss may have a considerable negative impact on the ability to participate in social activities, and may cause social isolation and reduce educational attainments and employment opportunities [16, 18, 31]. Even mild hearing loss can cause great difficulty in hearing and understanding another person talking in a noisy place, for instance on an urban street, or in a social setting with many people [16, 29]. Also those with a unilateral hearing loss may have difficulties in following or taking part in a conversation [29].

More than 80% of our study participants reported having had at least one episode of acute otitis during childhood, and almost half had a history of adenoidectomy. These findings are consistent with a recent US study by Okenfuss et al., where 74% of the participants reported recurrent episodes (≥ 3) of otitis media infection, and 40% had undergone adenoidectomy [11]. In a study by Ireland et al., 50% of the children had ventilation tubes inserted before the age of 3 years, consistent with 44% observed in our study [32].

The majority of those diagnosed with hearing loss in our study had conductive hearing loss, consistent with prior studies in achondroplasia [7, 11, 15]. Conductive hearing loss is commonly caused by middle ear effusion due to Eustachian tube dysfunction [5, 20], as was also reflected by the abnormal tympanometry (Type B or C) and stapedius reflex observed in over 70% of our study participants. Occupational noise exposure is another risk factor associated with hearing loss in adults [20]. However, none of the study participants reported prior exposure to occupational noise, so this is unlikely to explain the high prevalence of hearing loss in our study population.

In average statured persons, hearing loss is usually more prevalent in men than in women, across all age groups [17, 33]. In contrast, there was no considerably difference in hearing loss between men and women in our achondroplasia study population, likely because the abnormal craniofacial skull anatomy is present in both men and women with achondroplasia [3, 7].

While over half of the participants in our study had hearing loss, only 20% were currently using hearing aids. This proportion is comparable to studies conducted on average stature populations [20, 31]. In the general population, low confidence in effectiveness, high costs, lack of comfort, social norms, or cosmetic appearance are possible causes reported for the low use of hearing aids [20, 34]. These causes are probably applicable to adults with achondroplasia as well. Impaired hearing might also get less attention in achondroplasia due to the high prevalence of other potential severe medical complications, as well as the daily challenges commonly experienced by persons with this condition [10, 11, 24, 35–37].

### Clinical implications

The present study demonstrated a high prevalence of hearing loss in adults with achondroplasia. Of particular notice was the high prevalence of hearing loss observed in young persons with achondroplasia, consistent with prior studies [7, 9, 11]. These findings highlight the importance of a regular hearing assessment and adequate management and follow-up of acute and chronic middle ear disease during childhood in achondroplasia [10, 12, 23]. This should also include a low threshold for referral to an otolaryngologist in cases of recurrent otitis media, speech delay, or suspicion of chronic otitis media, in order to treat properly, and decrease the risk of permanent hearing loss [5, 12, 23, 38].

Our findings indicate that hearing loss might be underdiagnosed and undertreated in adults with achondroplasia, underlining the importance of a formal hearing assessment being conducted also in adolescents and young adults with this condition [23]. Further follow-up should be individualized, and with a low threshold for a subsequent hearing assessment if suspicion of impaired hearing is present.

Hearing loss may have a negative effect on work participation and educational achievements, and compensating strategies are strenuous and can cause tiredness [31]. In adult patients diagnosed with hearing loss, an evaluation by an otolaryngologist should be considered, and the need for hearing aids, assistive listening devices, and workplace and educational accommodations should be discussed. A variety of hearing aids and hearing assistive technology are available [20, 31, 34]. Also the acoustic environment and potential need for noise reducing measures should be considered, as well as flexibility regarding organizing the workday and tasks, how meetings and dialogues at work are organized, and the possibility of taking breaks when needed [31, 39]. This also applies to students at college or university, to facilitate optimal educational outcomes [40].

### Strengths and limitations

Strengths of this study are the population-based study sample, all having genetically confirmed achondroplasia, and a comprehensive audiologic assessment conducted in all participants. Other strengths are that all hearing assessments were conducted by an experienced investigator and in accordance with current international reference standards.

There are also limitations to this study. First, there were few participants in the oldest age group over 64 years of age. In the general population, hearing loss increases with age [17, 21].﻿﻿ As we probably have recruited a larger proportion of younger adults with achondroplasia in the present study, the overall prevalence rate of hearing loss in our study may be underestimated.

Second, the exact prevalence of achondroplasia within Norway is uncertain. We have estimated the population of adults (16 years or older) with achondroplasia in Norway to be between 66 and 101 adults [24]. As this study included somewhat fewer participants, there is a risk of selection bias that could have affected the present outcomes. However, except for few participants in the oldest age group, there was an even distribution regarding gender, age, and geographical residence in the study population.

F﻿inally, our study population consisted only of Caucasian participants, although this was not an inclusion criterion. Prevalence rates of hearing loss might be different in achondroplasia populations of other ethnicity, although the medical complication rates, including otitis media infections, seem to be relatively consistent across achondroplasia study populations of different geographical origin [9–11, 41, 42].

## Conclusions

This population-based study has demonstrated that adults with achondroplasia are at increased risk of early hearing loss, in contrast to the general population where hearing loss largely is caused by ageing. The high prevalence of early hearing loss underlines the importance of a regular hearing assessment being part of standard care in achondroplasia, including adolescents and young adults. In adult patients diagnosed with hearing loss, an evaluation by an otolaryngologist should be considered, and the need for hearing aids, assistive listening devices, and workplace and educational accommodations should be discussed. New precision therapies aimed at restoring normal endochondral ossification are now in final clinical development for children with achondroplasia [2, 43], and it will be interesting to see if these therapies decrease the incidence of hearing loss in this population.

## Data Availability

De-identified individual participant data are available from the corresponding author on reasonable request.
